# Intravenous injection of microvesicle-delivery miR-130b alleviates high-fat diet-induced obesity in C57BL/6 mice through translational repression of PPAR-γ

**DOI:** 10.1186/s12929-015-0193-4

**Published:** 2015-10-16

**Authors:** Shifeng Pan, Xiaojing Yang, Yimin Jia, Yue Li, Rirong Chen, Min Wang, Demin Cai, Ruqian Zhao

**Affiliations:** Key Laboratory of Animal Physiology & Biochemistry, Ministry of Agriculture, Nanjing Agricultural University, Nanjing, 210095 P. R. China; College of Veterinary Medicine, Yangzhou University, Yangzhou, 225009 P. R. China; Lab of Translational Medicine, Jiangsu Province Academy of Traditional Chinese Medicine, Nanjing, 210028 P. R. China

**Keywords:** MV-delivery miR-130b, PPAR-γ, Lipolysis, Epididymal fat deposition, High-fat diet induced obese mice

## Abstract

**Background:**

We have shown previously that microvesicle (MV)-delivered miR-130b (miR-130b-MV) is able to target PPAR-γ and subsequently reduce the lipid accumulation *in vitro*. However, the *in vivo* effect of miR-130b on fat deposition and glucose homeostasis remains unknown.

**Results:**

Three-week-old C57BL/6 mice were fed a high-fat diet for 8 weeks and then intravenously injected with MV-packaged scrambled control microRNA (miRNA) or miR-130b every other day for 10 days. Glucose tolerance test was performed and body weight, epididymal fat weight, as well as the expression of lipid metabolic genes were determined. We showed that mice fed on high-fat diet for 8 weeks demonstrated significantly higher body weight, elevated blood glucose and impaired glucose tolerance. miR-130b-MV injection significantly reduced body weight and epididymal fat weight and partly restored glucose tolerance. miR-130b expression was significantly increased in the epididymal fat after miR-130b-MV injection while the protein content of its target gene PPAR-γ was significantly suppressed, together with a significant up-regulation of the lipolysis genes, hormone sensitive lipase, monoglyceride lipase and leptin. Moreover, miR-130b-MV injection increased the expression of miR-378a and miR-378-3p that are reported to participate in the regulation of fat deposition.

**Conclusion:**

Our results indicate that miR-130b-MV is able to reduce the epididymal fat deposition and partly restore glucose tolerance, through translational repression of PPAR-γ in a high-fat diet-induced obese mouse model.

## Background

Obesity is a major risk factor for the development of type II diabetes (T2DM), cardiovascular diseases, and some types of cancer [1–5]. Searching for more efficient and safe approaches to prevent and treat obesity has been the focus of intensive research. Peroxisome proliferator activated receptor gamma (PPAR-γ) is a ligand activated transcription factor which is regarded as a master regulator of fat deposition [6–8]. Previous studies have shown that activation or over-expression of PPAR-γ can stimulate lipogenesis and adipogenesis [1], while down-regulation of PPAR-γ decreases fat mass in mice [9–13]. Therefore, PPAR-γ is considered as a therapeutic target for the treatment of obesity.

MicroRNAs (miRNAs) are small non-coding RNAs which regulate gene expression through mRNA degradation and/or translational repression [14]. Numerous studies have demonstrated that some miRNAs can inhibit PPAR-γ expression and suppress adipogenesis and lipogenesis. Both miR-302a and miR-27a are reported to inhibit adipogenic differentiation and lipid accumulation in 3T3-L1 mouse adipocytes [15] and human multipotent adipose-derived stem cells [16] by down-regulating PPAR-γ expression. miR-130 has been shown to strongly reduce adipogenesis by repressing PPAR-γ biosynthesis in human primary preadipocytes and 3T3-L1 mouse adipocytes [17]. These findings suggest that miRNAs may be used as an effective therapy for the treatment of obesity. However, exogenous miRNAs without appropriate protection or modifications can be quickly degraded by RNases that are abundant in the blood [18]. Therefore, the stability of exogenous miRNAs is one of the primary concerns with respect to clinical application [19].

Microvesicles (MVs) are a heterogeneous population of membrane-covered vesicles ranging from 100 nm to 1 μm in diameter, being secreted by almost all types of cells *in vivo* and *in vitro* under both normal and pathological conditions [20–23]. MVs are able to protect, transport and deliver bioactive contents, including miRNAs [24], from parent cells to cells of other origins [25, 26]. Recently, we demonstrated that miR-130b can be packaged into MVs and delivered to the recipient primary cultured porcine adipocytes to reduce lipid accumulation *in vitro* by inhibiting PPAR-γ expression [27] . Nevertheless, it remains unknown whether MV-shuttled miR-130b can modulate fat deposition through targeting PPAR-γ *in vivo*.

Therefore, in the present study, we tested anti-obesity efficacy of MV-packaged miR-130b on a high-fat diet-induced C57BL/6 mouse model. miR-130b was packaged into MV by HeLa-229 cells transfected to over-express exogenous miR-130b. MV-packaged miR-130b was isolated from the culture media through ultracentrifugation and was injected intravenously to the obese mice. We show that miR-130b was delivered to the epididymal fat tissue and significantly decreased the fat deposition, which was associated with a significant down-regulation of PPAR-γ protein content and an activation of lipolytic genes. Our results provide the preliminary evidences that MV-mediated delivery of miR-130b is able to reduce fat deposition in a high-fat diet-induced obese model.

## Methods

### Reagents, cells, and antibodies

Dulbecco's modified Eagle's medium: Nutrient Mixture F-12 (DMEM/F-12) was supplied by Life Technologies Inc. (Carlsbad, CA, USA). Fetal bovine serum (FBS) was obtained from HyClone (Logan, UT, USA). The human cervix cancer cell line HeLa-229 was purchased from the Cell Resource Center of Shanghai Institute for Biological Sciences, Chinese Academy of Sciences (Shanghai, China). Anti-PPAR-γ (BS4444, 1: 500 dilution) and anti-GAPDH antibodies (AP0066, 1: 10,000 dilution) were purchased from Bioworld Technology (Minneapolis, MN, USA). Synthetic RNA molecules and scrambled negative control oligonucleotides were purchased from Life Technologies Inc..

### Plasmid construction

The precursors of miR-130b (89 bp) and the negative control miRNA (Scrambled control, miR-SC) were synthesized by Life Technologies Inc., based on the sequence information from miRNA precursors (www.mirbase.org) and the requirements for p*Silencer* 3.1-H1 siRNA expression vector (Ambion, Austin, TX, USA). Precursors of miR-130b and miR-SC were produced by annealing the upstream and downstream (50 μmol/L each) miRNA precursor sequences (Table 1). The 50 μL reaction mix was incubated in 96-well plates at 95 °C for 2 min, and subjected to touchdown PCR. During this procedure the temperature was decreased 0.1 °C every 8 s until it reached 25 °C. The PCR products were subcloned into pSilencer 3.1-H1 siRNA expression vector using *BamHI* and *HindIII* restriction endonucleases (Life Technologies Inc.).

**Table 1:** Primers used for plasmids construction and mRNA quantification

Name	Sequence
Plasmids construction	
*ssc-miR-130b (MI0013136)*	F: GATCCGCCTGCCTGACACTCTTTCCCTGTTGCACTACTG
	TGGGCCACTGGGAAGCAGTGCAATGATGAAAGGGCATCA
	GTCAGGCTTTTTTGGAAA
	R: AGCTTTTCCAAAAAAGCCTGACTGATGCCCTTTCATCAT
	TGCACTGCTTCCCAGTGGCCCACAGTAGTGCAACAGGGA
	AAGAGTGTCAGGCAGGCG
*ssc-miR-SC*	F: GATCCGACTTACAGCCAGTTCCTAGTATAGTGAAGCAG
	CAGATGGTATACTAGGAACTGGCTGTAAGCTTTTTTTGGA
	AA
	R: AGCTTTTCCAAAAAAAGCTTACAGCCAGTTCCTAGTA
	TACCATCTGCTGCTTCACTATACTAGGAACTGGCTGTAAGTCG
mRNA expression	
*PPAR-*γ (NM_138711)	F: GCCCTTCACCACTGTTGATT
	R: GAGTTGGAAGGCTCTTCGTG
*GR* (AY779185)	F: CCAAACTCTGCCTTGTGTGTTC
	R: TGTGCTGTCCTTCCACTGCT
*TNF-*α (NM_013693.3)	F: CTATGGCCCAGACCCTC
	R: GCAGCCTTGTCCCTTGA
*UCP-3* (NM_009464.3)	F: ACGATGGATGCCTACAGGAC
	R: TCCGAAGGCAGAGACAAAGT
*LDLR* (NM_001252658.1)	F: TCAGTCCCAGGCAGCGTAT
	R: TGATCTTGGCGGGTGTT
*STAT3* (NM_011486.4)	F: ATTGTGATGCCTCCTTGA
	R: ATTGGCGGCTTAGTGAA
*FAS* (EF589048)	F: GTCCTGCTGAAGCCTAACTC
	R: TCCTTGGAACCGTCTGTG
*SCD-1* (NM_213781)	F: CCCAGCCGTCAAAGAGAA
	R: CGATGGCGTAACGAAGAAA
*11*β*-HSD1* (AF414124)	F: CCATGCTGAAGCAGAGCAAC
	R: AAGAACCCGTCCAGAGCAAA
*HSL* (AY686758)	F: ACCCTCGGCTGTCAACTTCTT
	R: TCCTCCTTGGTGCTAATCTCGT
*ATGL* (EF583921)	F: ACCTGTCCAACCTGCTGC
	R: GCCTGTCTGCTCCTTTATCCA
*MGL* (NM_001166249.1)	R: CATTGCTCGCTCCACTCTT
	F: ATGGTCCTGATTTCACCTCTG
*Leptin* (NM_008493.3)	F: CCCTCATCAAGACGATTGTCA
	R: GGTTCTCCAGGTCATTCGATA
*LeptinR* (NM_001122899.1)	F: CCCTCATCAAGACGATTGTCA
	R: GGTTCTCCAGGTCATTCGATA
*ACC* (NM_133360.2)	F: AGCAGTTACACCACATACAT
	R: TACCTCAATCTCAGCATAGC
*SREBP-1* (NM_011480.3)	F: GCTTCTCTTCTGCTTCTCT
	R: GCTGTAGGATGGTGAGTG
*PPIA* (NM_214353.1)	F: TCCTCCTTGGTGCTAATCTCGT
	R: TGATCTTCTTGCTGGTCTT

### Cell culture, miR-130b transfection and microvesicle isolation

Approximately 3 × 10^5^/cm^3^ HeLa-229 cells were seeded in 150 mm cell culture dish and grown in DMEM/F-12 media supplemented with 15 mmol/L NaHCO_3_, 100 IU/mL penicillin, 100 IU/mL streptomycin, and 10 % FBS at 37 °C in a 5 % CO_2_, water-saturated incubator. When the cells reached 90-95 % confluence, plasmids of 50 μg miR-130b and 50 μg miR-SC were transfected separately with Lipofectamine 2000 (Life Technologies Inc.), according to the manufacturer’s instructions. The transfected cells were incubated at 5 % CO_2_ and 37 °C. Four hours later, the transfection medium was changed to DMEM/F-12 containing 10 % MVs-free FBS prepared by ultracentrifugation and filtration [28]. Cells were harvested 24 h after transfection and the medium was collected.

MVs were isolated from the medium by differential centrifugation according to previously published methods [29]. Briefly, 18 mL media mixture from six dishes was subjected to serial centrifugation. Initial centrifugation was undertaken at 300 g for 10 min followed by 1200 g for 10 min and 10,000 g for 20 min, by this process dead cells and other debris were removed. Then the resulting supernatant was filtered through 0.22 μm filters (Millipore, Billerica, MA, USA) into Beckman Quick seal tubes. Ultracentrifugation was performed at 110,000 g for 2 h using a 70Ti rotor (Beckman Coulter, Brea, CA, USA). All steps were performed at 4 °C. MVs were collected from the pellets and re-suspended in FBS-free media for subsequent assay. The Bicinchoninic acid (BCA) method was used to quantify the total protein concentration in MVs preparations.

### Animals and diets

All procedures involving laboratory animal use were approved by the Animal Ethics Committee of Nanjing Agricultural University, with the project number 2012CB124703. The slaughter and sampling procedures complied with the “Guidelines on Ethical Treatment of Experimental Animals” (2006) No. 398 set by the Ministry of Science and Technology, China.

Three-week-old male specific pathogen-free (SPF) C57BL/6 mice weighing 9 ~ 10 g were obtained from the Comparative Medicine Center of Yangzhou University (Yangzhou, China, certificate of quality is SCXK (Su) 2012-0004) and fed in the Laboratory Animal Center of Jiangsu Province Integrative Medicine Hospital. The mice were housed in standard cages (33 × 23 × 12 cm, five mice/cage), maintained under controlled conditions (22 ± 0.5 °C, 50 ± 5 % relative humidity, 12-h/12-h dark/light cycle) with free access to both food and water.

After 7-day adaptation, thirty-six mice were randomly divided into two groups as follows: (1) the control group (MD10% fat group, *n* = 12) fed with normal fat diet (MD12031, 10 % fat); (2) the high fat group (MD45% fat group, *n* = 24) fed with high-fat diet (MD12032, 45 % fat). Both control and high fat diets were purchased from Medicience Ltd. (Yangzhou, China). The diets were replaced every 2 days to prevent oxidization of the fats in diets. After 8 weeks, the body weight was recorded and the glucose tolerance test was performed to confirm the successful establishment of the obese mouse model.

Then, we divided the obese mice randomly into two groups: (1) the control group injected with miR-SC-MV (HF-SC-MV); (2) the treated group injected with miR-130b-MV (HF-130b-MV). The mice were injected every other day for 10 days. During the 10 days of treatment, the mice in both groups were still fed high-fat diet.

### Oral glucose tolerance test (OGTT)

After 10 h fasting (from 8:30 in the morning till 18:30 in the afternoon), the mice were given glucose at 2.5 g/kg body weight by intraperitoneal injection. The blood glucose levels before glucose injection (0 min) and 15, 30, 60, 90, 120 min after glucose injection were determined.

### Preparation of blood and epididymal fat tissue

After ten days of treatment, the mice were fasted for 10 h and the body weight was recorded. Then the blood was drawn from the abdominal aorta using a syringe. The plasma was separated by centrifugation at 3000 rpm for 15 min at 4 °C and stored at -20 °C. The epididymal fat, gastrocnemius muscle and liver samples were removed, weighed and snap-frozen in liquid nitrogen and then stored at -70 °C.

### Analyses of plasma biochemical parameters, hormones and cytokines

Plasma concentrations of biochemical metabolites, including alanine transaminase (ALT, no.C009-2), aspartate transaminase (AST, no.C010-1), glucose (GLU, no.F006), triglycerides (TG, no.F001-1), total cholesterol (TCh, no.F002-1), high-density lipoprotein cholesterol (HDLc, no.A112-2), low-density lipoprotein cholesterol (LDLc, no.A113-1) and nonestesterified fatty acid (NEFA, no.A042-1) were detected by automatic biochemical analyzer (Beckman coulter, AU2700) using commercial kits (Jiancheng Bioengineering Institute, Nanjing, China). The plasma concentrations of hormones and cytokines, such as interleukin-6 (IL-6, no.96-407), insulin (no.96-416), leptin (no.96-421) and tumor necrosis factor (TNF-α, no.96-422), were measured by China Biomarker Service, Luminex 200 (no.CNBMSLX200) using Magnetic Bead MAPmate (Merck&Millipore, Darmstadt, Germany) according to the instructions provided by the manufacturer.

### RNA isolation and mRNA quantification

Total RNA was isolated from liver using Trizol reagent (Life Technologies Inc.), according to the manufacturer’s instructions. Concentration of the extracted RNA was measured using a NanoDrop-1000 spectrophotometer. RNA integrity was confirmed by denaturing agarose electrophoresis, and DNA contamination was evaluated by PCR using isolated RNA as template with the primers of 18s. M-MLV (Promega, Madison, WI, USA) and dN6 random primer (Takara, Kyoto, Japan) were used to synthesize cDNA from 2 μg of total RNA from each sample according to manufacturer’s instructions. Three reference genes (PPIA, GAPDH and 18s) were tested and the mRNA abundances showed no difference between the two groups, and at last PPIA was chosen as a reference gene. Real-time PCR was performed in Mx3000P (Stratagene, Palo Alto, CA, USA). All primers used for this experiment were listed in Table 1.

### miRNAs real-time PCR quantification

Total RNA was treated with RNase-free, DNase I (TaKaRa). The total RNA (4 μg) was polyadenylated by poly (A) polymerase at 37 °C for 1 h in a 20 μL reaction mixture using a Poly (A) tailing kit (AM1350, Applied Biosystems, Carlsbad, CA, USA) according to the manufacturer’s instructions. The polyadenylated RNA was then dissolved and reverse transcribed using the poly (T) adapter.

Real-time PCR was performed, in triplicate, using the SYBR green qPCR master mix reagent (Takara) with a miRNA-specific forward primer and a universal reverse primer that is complementary to part of the poly (T) adapter sequence. Since no validated reference gene was available for pig miRNAs, a random DNA oligonucleotide was added to RNase-free DNase I-treated total RNA samples before polyadenylation, as an exogenous reference, to normalize the expression of miRNAs. The sequences of all the mature miRNAs, the poly (T) adapter and the exogenous reference gene used in the present study are listed in Table 2.

**Table 2:** primers used for miRNA detection

Name	Primer sequence (5’ to 3’)	miRbase Acc No.
miR-130b	CAGUGCAAUGAUGAAAGGGCAU	MIMAT0013922
miR-130a	CAGUGCAAUGUUAAAAGGGCAU	MIMAT0007758
miR-27a	UUCACAGUGGCUAAGUUCCGC	MIMAT0002148
miR-27b	UUCACAGUGGCUAAGUUCUGC	MIMAT0013890
miR-103	AGCAGCAUUGUACAGGGCUAUGA	MIMAT0002154
miR-143-3P	UGAGAUGAAGCACUGUAGCUC	MIMAT0013879
miR-143-5P	GGUGCAGUGCUGCAUCUCUGG	MIMAT0017374
miR-378a	ACUGGACUUGGAGUCAGAAGGC	MIMAT0013868
miR-378b-3p	ACUGGACUUGGAGUCAGAAGU	MIMAT0037082
miR-455	UAUGUGCCUUUGGACUACAUCG	MIMAT0022957
miR-320	AAAAGCUGGGUUGAGAGGGCGAA	MIMAT0013878
miR-106a	AAAAGUGCUUACAGUGCAGGUAGC	MIMAT0002118
poly(T) adapter	TAGAGTGAGTGTAGCGAGCACAGAA	N/A
	TTAATACGACTCACTATAGGTTTTTT	
	TTTTTTTTTTVN	
Universal primer	TAGAGTGAGTGTAGCGAGCA	N/A
Exogenous reference	GTGACCCACGATGTGTATTCGC	

### Determination of PPAR-γ protein content

Total protein was extracted from the epididymal adipose tissue and the protein concentration was measured using a BCA protein assay kit (Pierce, Rockford, IL, USA) according to the manufacturer’s instructions. Protein extracts (30 μg) were used for electrophoresis on a 12 % SDS-PAGE gels. Western blot analysis for detecting PPAR-γ was undertaken according to the protocols provided by the manufacturer. GAPDH was used as a reference.

### Statistical analysis

All data are presented as means and standard errors (± SEM). For oral glucose tolerance test, the general linear model (univariate) was conducted to evaluate the effects of miR-130b-MV injection and the injection time point, as well as their interactions. The differences between two groups were tested using one-way analysis of variance (ANOVA). All statistical analyses were undertaken using Statistical Program for Social Sciences (SPSS) version 18.0 for Windows (SPSS Inc., Chicago, IL, USA). The level of significance was set at *P* < 0.05.

## Results

### Establishment of high-fat diet-induced obese mouse model

**Fig. 1:**
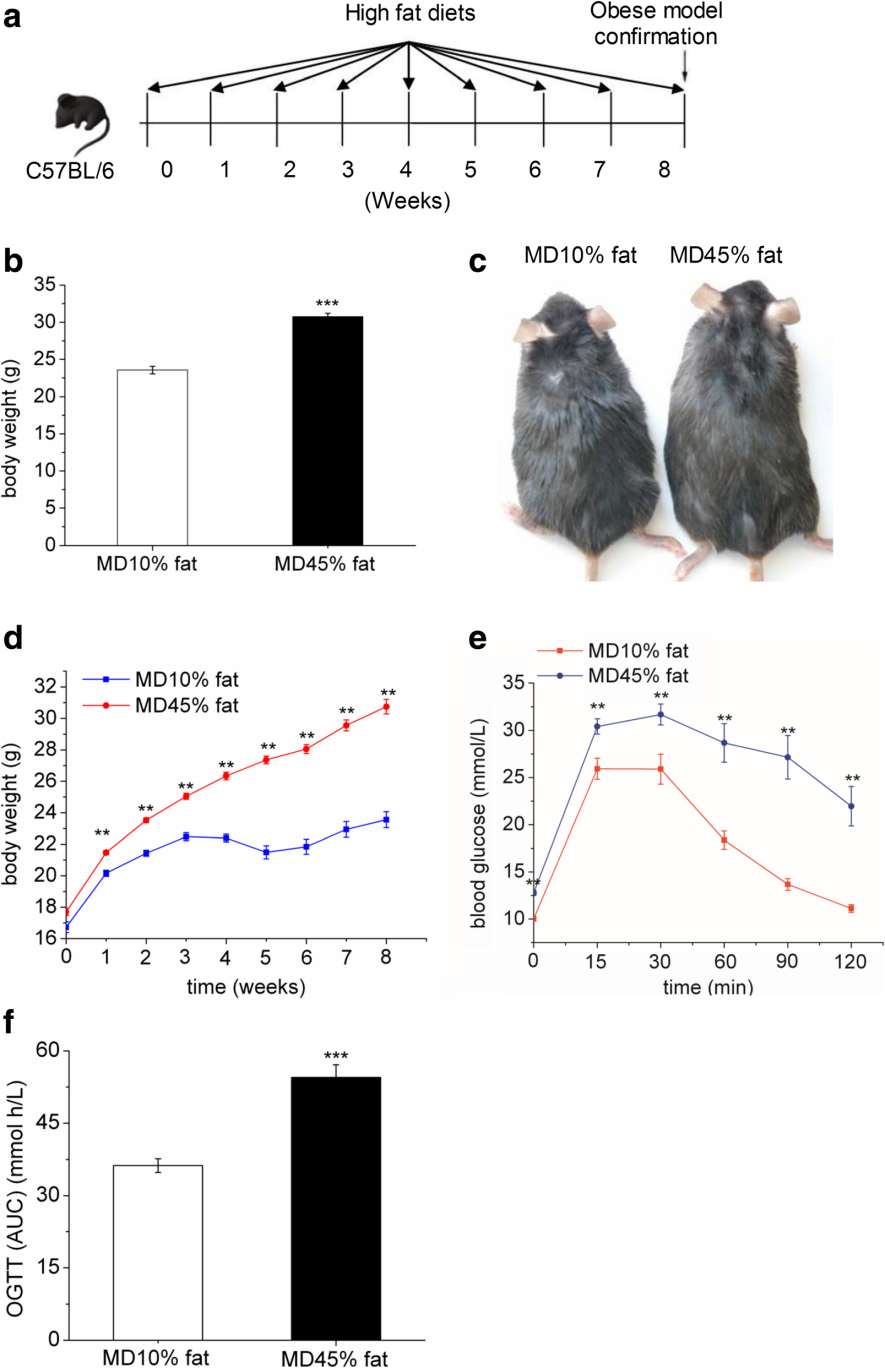
Establishment of high-fat diet-induced obese mouse model. **a**: Flow chart depicting the experimental design. Three-week-old C57BL/6 mice were fed with a normal fat diet (MD10% group) or a high fat diet (MD45% group) for 8 weeks. The obese mice model was confirmed by detecting body weight and OGTT. **b**: Body weight of C57BL/6 mice after feeding high-fat diet for 8 weeks. **c**: Phenotype of C57BL/6 mice. **d**: Changes in body weight in C57BL/6 mice during 8 weeks’ high-fat diet feeding. **e**: Mean blood glucose levels following OGTT. **f**: Area under the blood glucose-time curve (AUC). MD10%, 10 % fat medicience diet, normal fat diet group; MD45%, 45 % medicience diet, high-fat diet group; OGTT, oral glucose tolerance test; The values are presented as the means ± SEM, *n* = 12; *P < 0.05 *vs*. MD10% group; **P < 0.01 *vs*. MD10% group; ***P < 0.001 *vs*. MD10% group

C57BL/6 obese mouse model was successfully established after feeding high-fat diet ad libitum for 8 weeks (Fig. 1). The experimental design for establishing the obese mice model is depicted in a flow chart (Fig. 1a). The body weight was significantly higher (*P* < 0.01) in high-fat diet group (Fig. 1b-c) with significantly increased (*P* < 0.05) body weight gain (Fig. 1d). The oral glucose tolerance test (OGTT) demonstrated impaired glucose tolerance in high-fat diet group as compared with the control group (Fig. 1e), which was indicated by significantly increased (*P* < 0.001) area under the blood glucose-time curve (AUC) (Fig. 1f).

### miR-130b-MV partly restored glucose tolerance

**Fig. 2:**
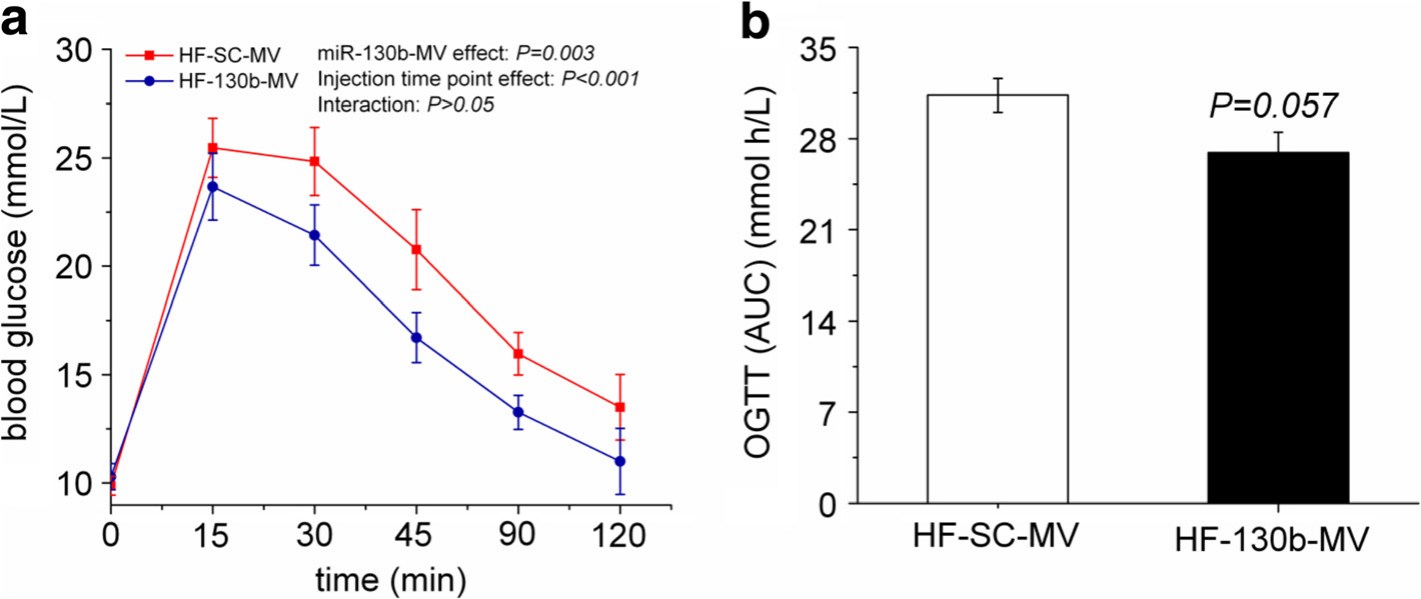
Mean blood glucose levels following OGTT. Mice were fasted 10 h and glucose was administered by intraperitoneal injection at 2.5 g/kg body weight. **a**: Mean blood glucose levels following OGTT. **b**: Area under the blood glucose-time curve (AUC). OGTT, oral glucose tolerance test; HF, high fat; HF-SC-MV, high-fat diet-induced obese mice injected with miR-SC-MV; HF-130b-MV, high-fat diet-induced obese mice injected with miR-130b-MV; The values shown represent the means ± SEM, *n* = 12; *P* < 0.01 *vs*. HF-SC-MV group

To determine the effects of miR-130b-MV injection on the glucose tolerance, OGTT was performed (Fig. 2). Blood glucose concentrations in miR-130b-MV-injected mice were significantly reduced (*P* = 0.003) after the intravenous injection of glucose (2.5 g/kg body weight) when compared with miR-SC-MV-injected mice (Fig. 2a). In addition, miR-130b-MV injection tended to reduce (*P* = 0.057) the AUC when compared with miR-SC-MV-injected mice (Fig. 2b). The above results suggest that intravenous injection of miR-130b-MV partly restored the glucose tolerance caused by high-fat diet.

### miR-130b-MV reduced body weight and epididymal fat weight

**Fig. 3:**
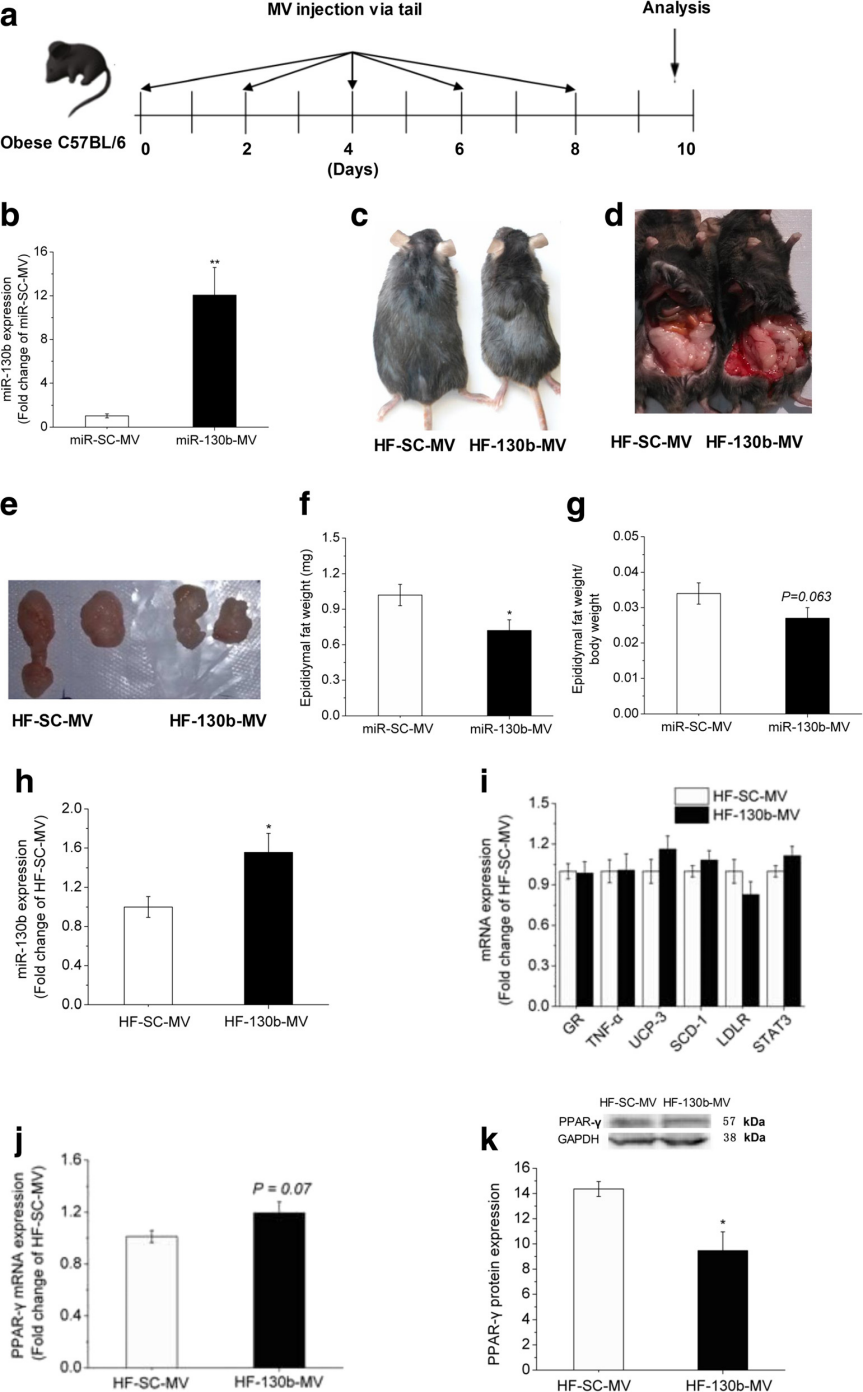
Epididymal fat deposition, miR-130b and its target gene PPAR-γ expression. **a**: Flow chart depicting the experimental design. The obese C57BL/6 mice were injected via tail vein with miR-SC-MV or miR-130b-MV every other day for 10 days. miR-130b-MV was harvested by transfecting HeLa-229 cells with miR-130b plasmid. Ten days later, the mice were weighed and sampled. **b**: Gene expression of miR-130b in miR-130b-MV. **c**: Body sizes. **d-e**: Epididymal fat sizes. **f-g**: Epididymal fat weight and the ratio between epididymal fat weight and body weight. **h**: Expression of miR-130b gene in the epididymal fat. **i**: mRNA expression of the miR-130b target genes. **j**: mRNA expression of the miR-130b target gene PPAR-γ. **k**: Protein expression of miR-130b target gene PPAR-γ. HF, high fat; HF-SC-MV, high-fat diet-induced obese mice injected with miR-SC-MV; HF-130b-MV, high-fat diet-induced obese mice injected with miR-130b-MV; The values shown represent the means ± SEM, *n* = 12; *P < 0.05 *vs*. HF-SC-MV group; **P < 0.01 *vs*. HF-SC-MV group

The protocol of MV injection via tail vein is depicted in the flow chart (Fig. 3a). We delivered miR-130b-MV to assess the anti-obesity effect of miR-130b *in vivo*. The HeLa-229 cell expresses very low level of miR-130b [27], so it was chosen to serve as a carrier host for the over-expression of exogenous miR-130b. HeLa-229 cells were transfected with the miR-130b over-expression plasmid, while plasmid over-expressing miR-SC was also transfected to serve as a negative control. MVs containing miR-130b and miR-SC were purified from the supernatant of transfected cells and RT-PCR verified the package of significantly higher levels of miR-130b in miR-130b-MV preparations (Fig. 3b). miR-130b-MV and miR-SC-MV preparations were injected via tail vein into the obese mice of treatment and control groups respectively every other day for 10 days. The phenotypic changes in body size and fat deposition are shown in Fig. 3c-e, which indicate obviously reduced body weight and the epididymal fat mass in mice treated with miR-130b-MV.

Quantitatively, miR-130b-MV significantly reduced the body weight (*P* < 0.01), the epididymal fat weight (*P* < 0.05) (Fig. 3f) and the muscle weight (*P* < 0.05), but not the liver weight. Furthermore, the epididymal fat weight relative to the body weight tended to be lower (*P* = 0.063) (Fig. 3g) in miR-130b-MV group when compared with miR-SC-MV group. The relative muscle weight was not different, yet the liver weight relative to the body weight was significantly higher (*P* < 0.05) in miR-130b-MV group compared with miR-SC-MV counterparts (Table 3).

**Table 3:** Effect of miR-130b-MV injection for 10 days on the apparent and blood biochemical parameters in mice

Parameters	HF-SC-MV	HF-130b-MV	*P* Value
Sampling parameters			
Body weight (g)	29.75 ± 0.53	26.30 ± 0.90	0.000
Epididymal fat weight (mg)	1.02 ± 0.09	0.72 ± 0.09	0.034
Liver weight (mg)	1.18 ± 0.05	1.17 ± 0.06	0.822
Muscle weight (mg)	0.34 ± 0.02	0.29 ± 0.01	0.025
Epididymal fat weight/Body weight	0.034 ± 0.003	0.027 ± 0.003	0.063
Liver weight/Body weight	0.039 ± 0.002	0.045 ± 0.002	0.004
Muscle weight/Body weight	0.011 ± 0.005	0.011 ± 0.005	0.825
Biochemical parameters			
ALT (U/L)	10.83 ± 1.72	10.45 ± 1.84	0.882
AST (U/L)	53.64 ± 5.68	52.50 ± 2.00	0.858
GLU (mmol/L)	11.92 ± 0.60	10.13 ± 0.74	0.074
TG (mmol/L)	0.55 ± 0.04	0.44 ± 0.04	0.109
Tch (mmol/L)	2.59 ± 0.13	2.41 ± 0.14	0.334
HDLc (mmol/L)	1.46 ± 0.08	1.33 ± 0.05	0.167
LDLc (mmol/L)	0.15 ± 0.02	0.20 ± 0.03	0.066
NEFA (μmol /L)	857.67 ± 41.17	879.82 ± 56.14	0.751
Hormones and cytokines			
IL-6 (pg/mL)	69.94 ± 14.17	216.98 ± 96.37	0.163
Insulin (pg/mL)	1668.54 ± 243.60	1618.35 ± 223.44	0.881
Leptin (pg/mL)	2566.43 ± 421.64	2289.77 ± 550.37	0.698
TNF-α (pg/mL)	6.66 ± 0.48	7.81 ± 0.69	0.194

Notes: HF, high fat; HF-SC-MV, high fat diet induced obese mice injected with miR-SC-MV; HF-130b-MV, high fat diet induced obese mice injected with miR-130b-MV; The values shown represent the means ± SEM; *P* < 0.05 means significant difference compared with the HF-SC-MV group; *n* = 12

miR-130b-MV treatment did not affect the plasma concentrations of ALT, AST, Tch, HDLc, LDLc, IL-6, insulin, leptin or TNF-α, while TG (*P* = 0.109) and glucose (*P* = 0.074) concentrations tended to be lower in miR-130b-MV group (Table 3).

### miR-130b-MV increased miR-130b expression and suppressed PPAR-γ protein content in epididymal fat

The abundance of miR-130b in the epididymal fat tissue was significantly higher (*P* < 0.05) in HF-130b-MV group (Fig. 3h). The mRNA expression of the predicted target genes of miR-130b, including GR, TNF-α, UCP-3, SCD-1, LDLR, STAT3, and PPAR-γ (Fig. 3i), was detected and only PPAR-γ tended to be increased (*P* = 0.07, Fig. 3j) in miR-130b-MV group. However, the protein content of PPAR-γ was significantly reduced (*P* < 0.05) in the epididymal fat of mice injected with miR-130b-MV (Fig. 3k).

### miR-130b-MV affected mRNA expression of lipid metabolic genes in epididymal fat

**Fig. 4:**
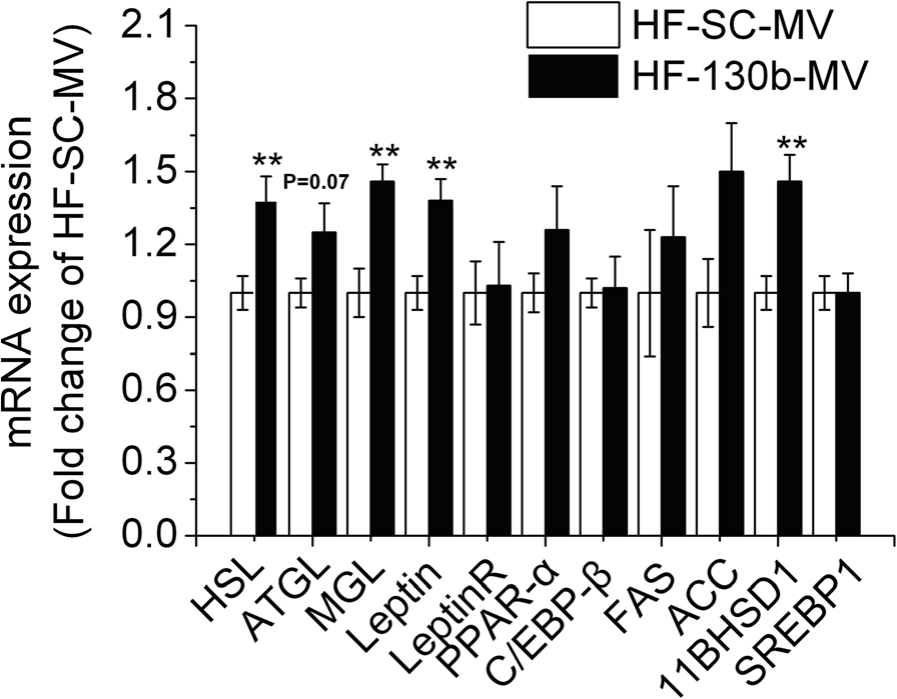
qRT-PCR analysis of PPAR-γ related genes in the epididymal fat of mice. HF, high fat; HF-SC-MV, high-fat diet-induced obese mice injected with miR-SC-MV; HF-130b-MV, high-fat diet-induced obese mice injected with miR-130b-MV; The values shown represent the means ± SEM, *n* = 12; *P < 0.05 *vs*. HF-SC-MV group; **P < 0.01 *vs*. HF-SC-MV group

Injection of miR-130b-MV affected the expression of lipid metabolic genes in the epididymal fat of high-fat diet-induced obese mice (Fig. 4). Hormone sensitive lipase (HSL) and monoacylglycerol lipase (MGL), the two lipolytic enzymes, as well as leptin, the appetite suppressing adipokine, were significantly up-regulated (*P* < 0.01) in the epididymal fat of high-fat diet-induced obese mice. The mRNA expression of adipose triglyceride lipase (ATGL) tended to be higher (*P* = 0.07) in miR-130b-MV-injected mice while the mRNA expression of leptin receptor was not affected.

Moreover, miR-130b-MV did not affect the mRNA expression of CCTTA enhancer binding protein-β (C/EBP-β), peroxisome proliferators-activated receptor-α (PPAR-α), sterol regulatory element-binding protein 1 (SREBP1), or the lipogenic enzymes, fatty acid synthase (FAS) and acetyl-CoA carboxylase (ACC). However, 11β-hydroxysteroid dehydrogenase type 1 (11β-HSD1) mRNA expression was significantly up-regulated (*P* < 0.01) in miR-130b-MV-injected mice when compared with miR-SC-MV mice (Fig. 4).

### miR-130b-MV affected expression of other miRNAs involved in lipid metabolism in epididymal fat

**Fig. 5:**
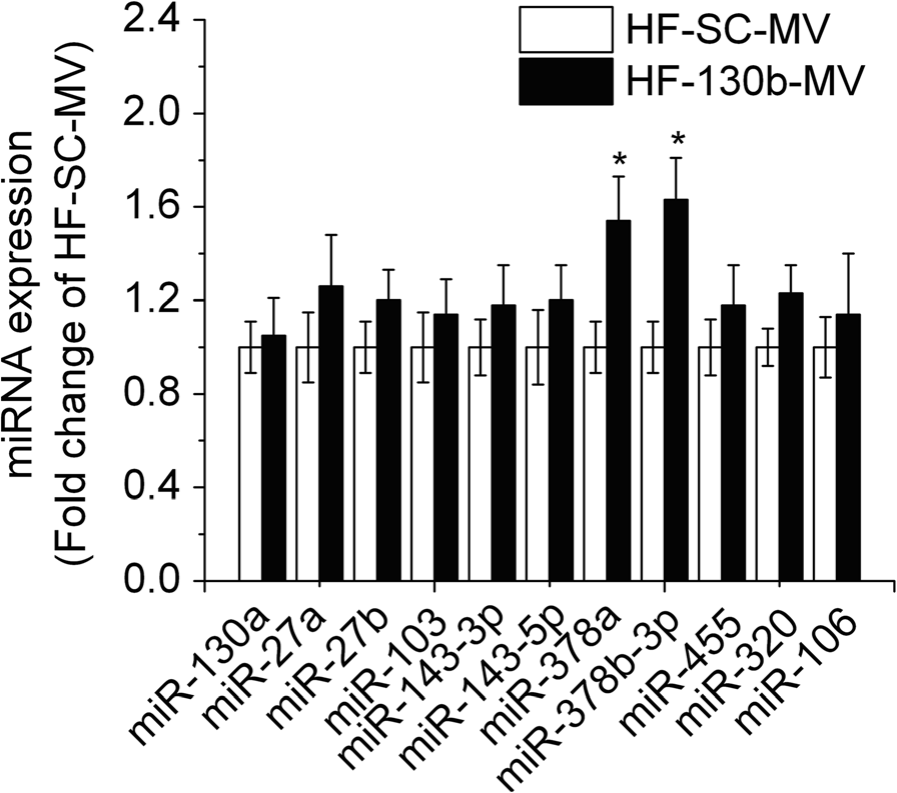
qRT-PCR analysis of fat deposition related miRNAs expression in epididymal fat of mice. HF, high fat; HF-SC-MV, high-fat diet-induced obese mice injected with miR-SC-MV; HF-130b-MV, high-fat diet-induced obese mice injected with miR-130b-MV; The values shown represent the means ± SEM, *n* = 12; *P < 0.05 *vs*. HF-SC-MV group; **P < 0.01 *vs*. HF-SC-MV group

The abundance of 11 other miRNAs in the epididymal fat that are closely related to fat deposition was also determined. The expression of miR-378a and miR-378b-3P was significantly increased (*P* < 0.05) in the epididymal fat of miR-130b-MV-injected mice when compared with miR-SC-MV-injected mice (Fig. 5).

## Discussion

High-fat induced obese C57BL/6 mice have been widely used as a model for studying human visceral obesity because they represent the human simple obesity both metabolically and pathophysiologically [30–32] . In this study, feeding C57BL/6 mice with high-fat diet for 8 weeks significantly increased the body weight by over 20 %, indicating that the obese mice model was successfully established [33]. Furthermore, blood glucose concentration was also significantly increased, suggesting impaired glucose tolerance which is closely associated with obesity [34].

In our previous study, we demonstrated that miR-130b-MV was able to reduce the lipid deposition in porcine primary adipocytes *in vitro* by targeting PPAR-γ gene [27]. Here we provide the evidence that miR-130b-MV injection was effective to decrease body weight and reduce epididymal fat deposition in high-fat diet-induced obese mice *in vivo*, at least partly through the translational repression of PPAR-γ. This is in agreement with a previous observation that miR-150-MV injection suppressed its target c-Myb expression and enhanced cell migration in mice [35] . Similarly, it was reported that miR-143-MV injection via tail vein was able to suppress tumor growth in mice [36]. Although there have been several attempts to administer MV-shuttled miRNAs by intravenous injection, this study provides, to our knowledge, the first evidence that miRNA packaged in MVs has an anti-obesity efficacy in an *in vivo* animal model.

However, the safety of miR-130b-MV-mediated therapy for obesity has to be considered. In the present study, we inspected histologically the possible side effects of miR-130b-MV on other tissues, including liver, kidney, heart and spleen. No obvious pathological changes were observed (H&E results not shown). Based on the consideration that each miRNA may target multiple target genes and function through different pathways [37], we detected numerous biochemical and hormonal parameters in the plasma including ALT, AST, Tch, TG, glucose, HDLc, LDLc, IL-6, insulin, leptin, and TNF-α. Interestingly, none of these blood parameters showed significant change, except TG and glucose displayed a tendency of decrease. This indicates that the general metabolic homeostasis of the body was not disturbed by miR-130b-MV treatment. Moreover, GR, TNF-α, UCP-3, SCD-1, LDLR, and STAT3, in addition to PPAR-γ, are also predicted to be the target of miR-130b, yet the mRNA expression of these genes in the epididymal fat tissue was not affected by miR-130b-MV treatment. Nevertheless, it remains to be determined whether these genes are affected at the level of protein.

We further investigated the down-stream molecular mechanisms underlying the miR-130b-MV-mediated inhibition in fat deposition. FAS and ACC are key adipogenic enzymes [38] that play pivotal roles in fat deposition, while HSL, ATGL and MGL are important lipases responsible for TG hydrolysis [39, 40]. Leptin, a cytokine secreted predominantly from the fat tissue, plays an important role in regulating energy balance, and increased leptin can stimulate lipolysis, by up-regulating HSL, ATGL and MGL expression [41]. In the present study, miR-130b-MV increased HSL, MGL and leptin mRNA expression, but did not influence FAS and ACC mRNA expression. Therefore, it is presumed that miR-130b-MV decreases fat deposition predominantly by enhanced lipolysis but not lipogenesis.

It is noted that miR-130b-MV also altered the expression of other miRNAs related to fat deposition. For instance, miR-378a and miR-378b-3p were up-regulated significantly in the epididymal fat tissue of miR-130b-MV-injected mice. miR-378 is highly induced during adipogenesis and has been reported to be positively regulated in adipogenesis. The role of miR-378 family in fat deposition has been controversial. Over-expression of miR-378 was shown to increase fat accumulation [42], yet the opposite result was also reported [43]. Over-expression of miR-378 increased lipolysis genes expression, while inhibition of miR-378 expression attenuated stimulated lipolysis and reduced the expression of lipolytic regulators [44]. MVs have been utilized for the delivery of therapeutic RNAi and are considered as a more effective, advantageous method than other options. Compared with the delivery strategies of viruses, lipid nanoparticles and polymeric nanoparticles, MVs present some major advantages. MVs are natural carriers and are not subjected to the attacks by antibodies, complements or opsonins in circulation. Other methods are prone to be cleared or trigger unwanted immune responses [45]. In the present study, MVs delivered miR-130b into the epididymal fat tissue efficiently and repressed the fat deposition, further suggesting that MVs are advantageous carriers for transferring therapeutic small RNAs compared to other methods. However, miR-130b-MV injection was conducted every other day for 10 days due to limited quantity of the miR-130b-MV preparations. The effects and the side-effects, if any, of prolonged treatment of miR-130b-MV remain unclear. It was reported that 7 days after miR-150b-MV injection, miR-150 still maintained at a low level in plasma, and its target gene VEGF was repressed and the tumor development was also suppressed [46]. Future studies are required to test the half-life of MV-protected miR-130b.

## Conclusion

In summary, this is the first *in vivo* study demonstrating that miR-130b-MV can be shuttled into the epididymal fat tissue to down-regulate PPAR-γ expression and to stimulate the expression of lipolysis genes. Further studies may be directed to assess the cytotoxicity and the half-life of miR-130b-MV in the blood, so as to further contribute to the development of anti-obesity drugs for clinical application.

## Abbreviations

ACC: Acetyl-CoA carboxylase
ALT: Alanine transaminase
AST: Aspartate transaminase
ATGL: Adipose tissue triglyceride lipase
ANOVA: Analysis of variance
BCA: Bicinchoninic acid
C/EBP-β: CCTTA enhancer binding protein-β
DMEM/F-12: Dulbecco's modified eagle's medium: nutrient mixture F-12
FAS: Fatty acid synthase
FBS: Fetal bovine serum
GAPDH: Glyceraldehyde-3-phosphate dehydrogenase
GLU: Glucose
GR: Glucocorticoid receptor
HDLc: High-density lipoprotein cholesterol
HF: High fat
HF-SC-MV: High fat diet-induced obese mice injected with miR-SC-MV
HF-130b-MV: High fat diet-induced obese mice injected with miR-130b-MV
HMEC-1: Human microvascular endothelial cell line-1
HSL: Hormone sensitive lipase
IBMX: 3-isobutyl-1-methylxanthine
IL-6: Interleukin-6
LDLc: Low-density lipoprotein cholesterol
MD: Medicience diet
MGL: Monoglyceride lipase
miRNAs: microRNAs
MV: Microvesicle
NEFA: Nonestesterified fatty acid
OGTT: Oral glucose tolerance test
PPAR-α: Peroxisome proliferators-activated receptor-α
PPAR-γ: Peroxisome proliferators-activated receptor-γ
PPIA: Peptidylprolyl isomerase A
qRT-PCR: quantitative reverse transcription polymerase chain reaction
SCD-1: Stearoyl-coenzyme A desaturase Type-1
SPF: Specific pathogen-free
SPSS: Statistical program for social sciences
SREBP1: Sterol regulatory element-binding protein 1
TCH: Total cholesterol
T2DM: Type II diabetes
TG: Triglycerides
THP-1: Human acute monocytic leukemia cell line-1
TNF-α: Tumor necrosis factor-α
WB: Western blotting
11β-HSD1: 11β-hydroxysteroid dehydrogenase type 1
